# Difficulty in Fixation of the Volar Lunate Facet Fragment in Distal Radius Fracture

**DOI:** 10.1155/2017/6269081

**Published:** 2017-01-31

**Authors:** Hiroyuki Obata, Tomonori Baba, Kentaro Futamura, Osamu Obayashi, Atsuhiko Mogami, Hideki Tsuji, Yoshiaki Kurata, Kazuo Kaneko

**Affiliations:** ^1^Department of Orthopaedic Surgery, Juntendo University Shizuoka Hospital, Shizuoka, Japan; ^2^Department of Orthopaedic Surgery, Juntendo University School of Medicine, Tokyo, Japan; ^3^Orthopaedic Trauma Center, Sapporo Tokushukai Hospital, Sapporo, Japan

## Abstract

Recent reports suggest the presence of a rare fracture type for which reduction and fixation cannot be achieved with volar locking plate (VLP). In particular, it is difficult to achieve reduction and fixation with volar lunate facet (VLF) fragments present on the volar ulnar aspect of the lunate facet, because of the anatomical structure and biomechanics in this region. Herein, we report two challenging cases of difficulty in fixation of the VLF fragment in distal radius fracture. For this fracture type, it is most important to identify the volar ulnar bone fragment before surgery; it may also be necessary to optimize distal placement of the VLP via a dual-window approach and to apply additional fixations, such as a small plate, anchor, and/or external fixation.

## 1. Introduction

Volar locking plate (VLP) fixation has become the gold standard for surgical treatment of distal radius fracture over the last 10 years because of its favorable postoperative outcomes. However, the presence of a rare fracture type has recently been suggested, in which a fragment is present on the volar ulnar aspect and reduction and fixation are difficult with VLPs. In 2004, Harness et al. reported that fixation of the volar lunate facet (VLF) fragment with a VLP is difficult because of the shape of the bone, which is flat on the sagittal view and slopes towards the ulnar side on the axial view [1]. Furthermore, in 2014 Beck et al. reported the risk factors for displacement of a VLF fragment based on various plain radiography parameters [2]. Reduction and fixation of a VLF fragment in distal radius fracture with VLPs are difficult anatomically (bone shape, approach) and biomechanically (distraction by ligaments) [1]. Reports focusing on VLF fragments have occasionally been published [2–4]. However, because this fracture type is very rare, no consensus has been reached on the definition of the fracture type, risk factors for displacement, or appropriate reduction and fixation methods. We encountered two patients with distal radius fracture with a VLF fragment and report these cases with a review of the literature.

## 2. Case Presentation

**Figure 1:**
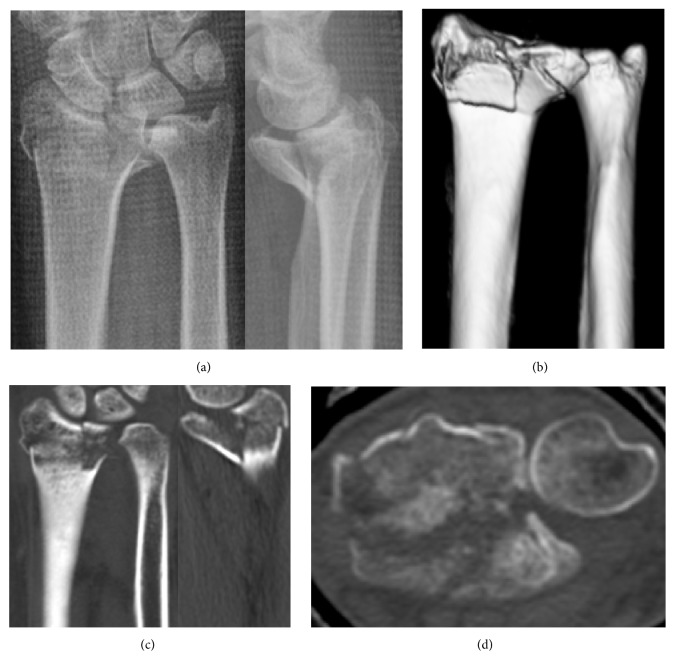
Patient 1. Plain radiographs (a) and CT (b, c, d) at the time of injury. A 10 × 8 mm VLF fragment is observed, with volar displacement.

**Figure 2:**
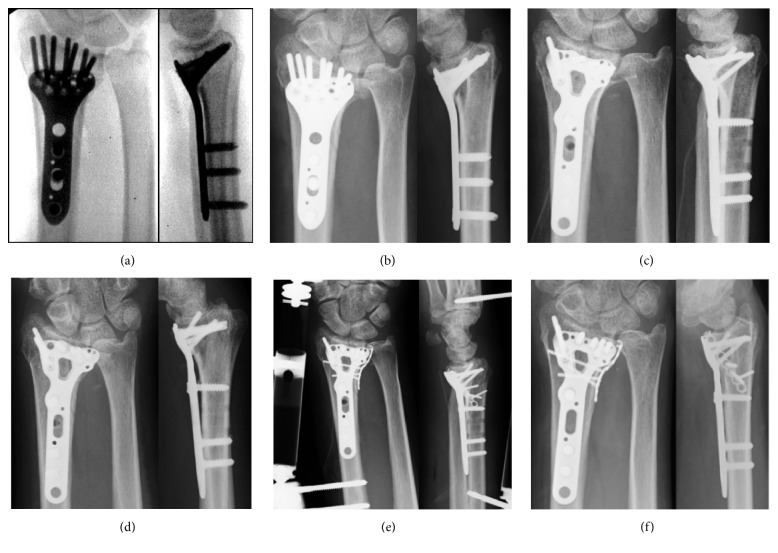
Plain radiographs of Patient 1. (a) Immediately after the first surgery. (b) Two weeks after the first surgery; redisplacement is seen. (c) Immediately after reoperation. (d) Five days after reoperation; redisplacement is seen. (e) Immediately after the third surgery. (f) One year after the third surgery; bone union was achieved without redisplacement.

*Patient 1*. A 54-year-old woman presented with right distal radius fracture (AO classification 23C3.1). A 10 × 8 mm VLF fragment was observed on plain radiography, with volar displacement (Figure 1). In the first surgery, reduction and fixation were achieved with a MODE VLP for proximal placement (Medical Dynamic Marketing Inc., Tokyo, Japan), used as a buttress plate (Figure 2(a)). However, redisplacement was noted 2 weeks after surgery (Figure 2(b)). At reoperation via a dual-window approach, reduction and fixation were achieved again with an Acu-Loc 2 VLP for distal placement (Acumed Co., Oregon, USA) (Figure 2(c)). However, redisplacement was again noted 5 days after surgery (Figure 2(d)). At second reoperation via a dual-window approach, the displaced VLF fragment was reduced from the ulnar side and fixed with a VariAx handplate (Stryker Co., Michigan, USA) used as a buttress plate; concomitant external fixation was applied for 4 weeks (Figure 2(e)). At 1 year after surgery, bone union was achieved without redisplacement (Figure 2(f)).

**Figure 3:**
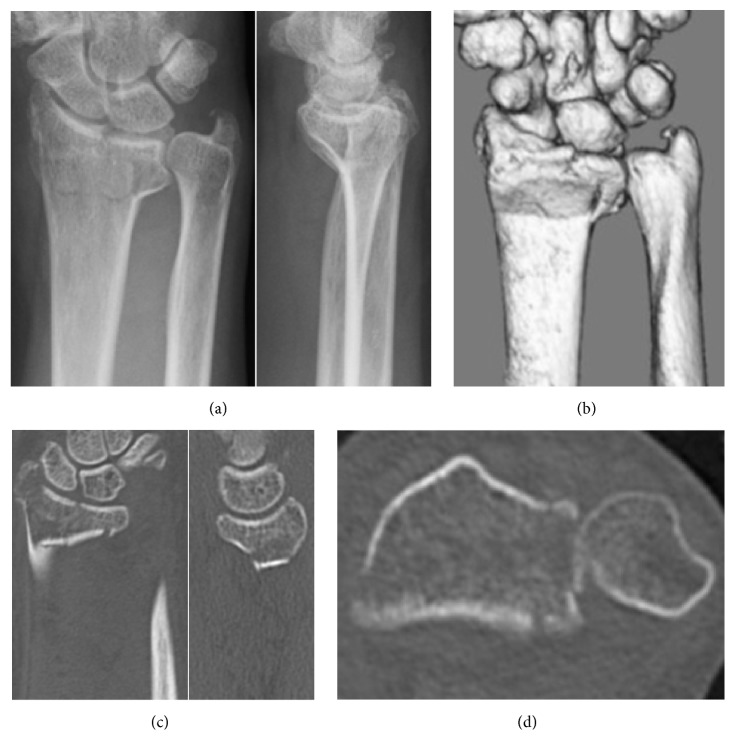
Patient 2. Plain radiographs (a) and CT (b, c, d) at the time of injury. A 12 × 12 mm VLF fragment is observed, with volar displacement.

**Figure 4:**
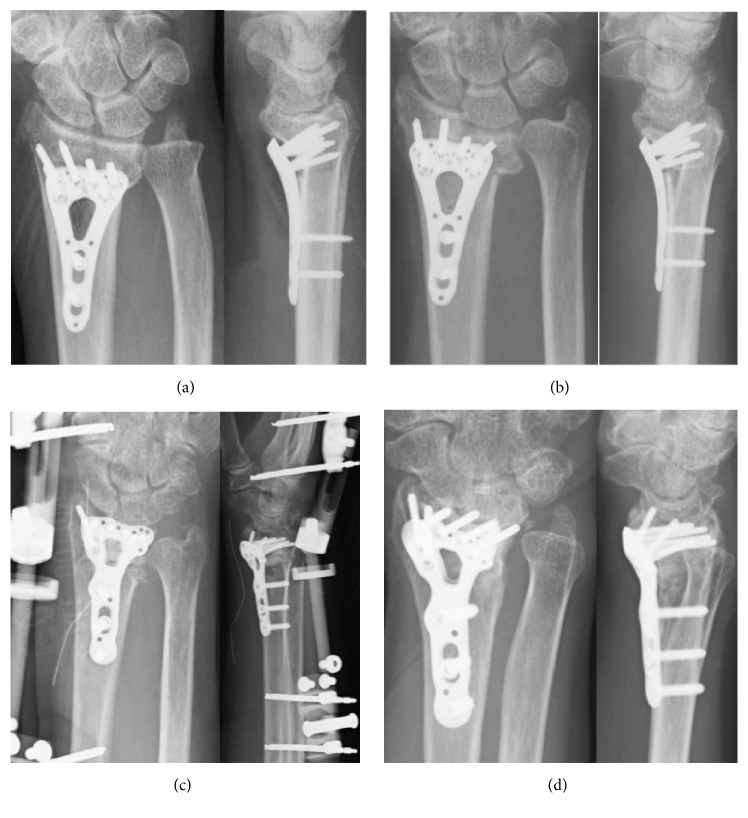
Plain radiographs of Patient 2. (a) Immediately after the first surgery. (b) Six weeks after the first surgery; redisplacement is seen. (c) Immediately after reoperation. (d) Nine months after reoperation; bone union was achieved without redisplacement.

*Patient 2*. A 58-year-old woman presented with right distal radius fracture (AO classification 23C3.1). A 12 × 12 mm VLF fragment was observed on plain radiography, with volar displacement (Figure 3). In the first surgery, reduction and fixation were achieved with a VA-TCP VLP for proximal placement (Depuy Synthes Co., Zuchwil, Switzerland), used as a buttress plate (for the distal screw, monoaxial screws were inserted over the guide block); however, redisplacement was noted after surgery (Figures 4(a) and 4(b)). Because 10 weeks had passed at the time of reoperation and malunion of the displaced VLF fragment was observed, corrective osteotomy was performed, followed by reduction and fixation which were achieved with an Acu-Loc 2 VLP for distal placement (Acumed Co., Oregon, USA) (Figure 4(c)). Concomitant external fixation was applied for 5 weeks. At 9 months after surgery, bone union was achieved without redisplacement (Figure 4(d)).

## 3. Discussion

The VLF fragment was first described by Harness et al. in 2004 [1]. That study reported difficulty in reduction and fixation of the VLF fragment with a VLP because of the anatomical structure and biomechanics in this region. In 2014 Beck et al. reported the risk factors for displacement of VLF fragments based on various plain radiography parameters [2]. However, plate coverage of the VLF fragment and the number of inserted screws were not associated with risk of displacement, suggesting the presence of a rare fracture type for which reduction and fixation cannot be achieved with VLPs. There have been several reports of reduction and fixation of VLF fragments [2–5], redisplacement resulting from inappropriate early treatment because of a lack of preoperative understanding of the VLF fragment. Because it is very rare for redisplacement with appropriate plate selection and positioning, it is difficult to objectively evaluate risk factors (e.g., size, shape, and position of the fragment) for VLF fragment displacement and to determine appropriate reduction and fixation methods. Many VLPs have been designed to provide double-tiered subchondral support: their structures support the dorsal subchondral bone at the center of the joint surface, because supporting the volar subchondral bone with screws is difficult [6]. Our first choice of VLP is the monoaxial mode, since we think that the stability of the monoaxial mode is higher than the polyaxial one. Both cases were stabilized by the monoaxial mode and it was possible for them to cover the lunate facet fragment by preoperative template. But support with a plate may also not be possible when the VLF fragment is small. On 3DCT, when a VLF fragment is present in the region contained within a straight line connecting the volar radial margin of the lunate facet and the proximal end of the distal radioulnar joint and the center of the distal radioulnar joint, instability occurs towards the volar ulnar side because the VLF fragment provides the attachment sites for the short radiolunate ligament and the distal radioulnar ligament. Of 177 cases of distal radius fracture treated surgically at our hospital, VLF fragment was present in five patients; the AO classification was C3 in each case. Two had volar displacement and three had dorsal displacement. Two had redisplacement after surgery. Both of these cases had volar displacement, and the fracture type was relatively simple, with mild crushing of the joint surface. Loads on the VLF fragment may be dispersed in fractures with marked joint surface crushing, whereas loads may be concentrated in relatively simple fractures with less joint surface crushing. In addition, loads may be more markedly concentrated in cases of volar displacement. The following characteristics may be risk factors for displacement: (1) a VLF fragment is present, (2) the joint surface is less crushed, and (3) the displacement is volar.

Regarding the reduction and fixation method, additional fixation with a miniscrew, K wire, soft wire, and hook plate has been reported [3]. However, these methods are likely to provide insufficient fixation force and to result in refracture. Therefore, when instability is marked and concomitant external fixation is applied it may be appropriate to apply a suture to the articular capsule with a small buttress plate and anchor as a basic procedure. The conventional trans-FCR approach can visualize VLF fragment adequately. However, when we have to place the buttress plate on the juxta-DRUJ, dual-window approach allows for the procedure of the buttress plating. This procedure cannot be achieved by trans-FCR approach. Stryker hand plating system was placed in bone slope towards DRUJ, so the edge of this small plate would not extend to the joint surface of DRUJ actually. Moreover, intraoperatively we confirmed pronation and supination of the forearm smoothly without the impingement of the implant. However postoperatively the limitation of supination occurred, so the placement of this small plate would cause some kind of factor (e.g., the adhesion of the soft tissue, the bulkiness of the plate). In fact, the limitation of the supination improved by the implant removal. Thus, if the limitation of supination and pronation occurred, we would have to remove the implant.

Redisplacement occurred in the current patients because a lack of preoperative understanding of the volar ulnar bone fragment resulted in inappropriate plate selection. Both cases were operated on in the different institutions. So the criteria for use of VLP differed in each institution. Redisplacement occurred despite optimal distal VLP placement, suggesting that reduction and fixation with available VLPs alone are difficult and that additional fixations are necessary, such as a small plate and external fixation. For fractures with a volar lunate facet fragment, it is necessary to identify the volar ulnar bone fragment. In the case of small volar lunate facet fragment, we suture the ligament on to the plate hole by using the anchor. In case of relative large volar lunate facet fragment, we think the small plate as a buttress plate from volar ulnar side is useful. As to the indication for the external fixator, if the stabilization of the internal fixation may be not enough, we use it in order to avoid the redisplacement. Both cases were operated on multiple times for the displacement of the volar lunate facet fragment. We had to immobilize the wrist joint by using the external fixation system in order to avoid the redisplacement.

For fractures with a VLF fragment, it is necessary to (1) identify the VLF fragment, (2) optimally place a VLP for distal placement via a dual-window approach, and (3) apply additional fixation (small plate, anchor, and/or external fixation).
